# Editorial: X-raying zero hunger (SDG2) targets in Africa and other regions: progress, synergies, opportunities, and challenges

**DOI:** 10.3389/fpubh.2024.1491501

**Published:** 2024-09-24

**Authors:** Olutosin Ademola Otekunrin, Mojisola Olanike Kehinde, Oluwaseun Ariyo, Barbara Sawicka

**Affiliations:** ^1^Innovation Lab for Policy Leadership in Agriculture and Food Security (PiLAF), University of Ibadan, Ibadan, Nigeria; ^2^Department of Agriculture, Landmark University, Omu Aran, Kwara, Nigeria; ^3^Department of Human Nutrition and Dietetics, University of Ibadan, Ibadan, Nigeria; ^4^Department of Plant Production Technology and Commodities Science, University of Life Science in Lublin, Lublin, Poland

**Keywords:** SDG2, food insecurity, malnutrition, nutrition security, food access, Africa

## 1 Introduction

Feeding a global population exceeding 8 billion people poses a monumental challenge, particularly for developing nations. The 2024 State of Food Security and Nutrition (SOFI) report paints a sobering picture: between 713 and 757 million individuals experienced hunger in 2023—a staggering one in eleven individuals worldwide, and one in five in Africa alone (1). This translates to a staggering 28.9% of the global population, or 2.33 billion people, facing moderate or severe food insecurity (1).

The convergence of the COVID-19 pandemic, the Russia-Ukraine conflict, climate change, poverty, growing inequalities, and soaring food prices has fueled a global hunger crisis, with Africa bearing the brunt. Africa faces the highest rate of hunger, with 20.4% of its population affected, compared to 8.1% in Asia, 6.2% in Latin America and the Caribbean, and 7.3% in Oceania (1–3). By 2030, an estimated 582 million people will be chronically undernourished, over half residing in Africa (1). Furthermore, both South Asia and sub-Saharan Africa recorded the highest hunger levels in 2023, with a Global Hunger Index (GHI) score of 27.0 each, highlighting the significant prevalence of hunger in these regions (3, 4).

Despite these grim realities, the United Nations (UN) remains committed to ending hunger through Sustainable Development Goal 2 (SDG 2), also known as Zero Hunger. This ambitious goal aims to “end hunger, achieve food security and improved nutrition, and promote sustainable agriculture” by 2030 (5). Since its launch in 2015, many nations have implemented food and nutrition-sensitive policies aimed at achieving this goal. However, persistent challenges, including poverty, population growth, conflict, and climate change, have hindered progress, particularly in Africa (6, 7).

This Research Topic aims to assess progress made in combating hunger globally, analyse the interconnectedness between SDG 2 (Zero Hunger) and other SDGs, and explore the potential opportunities and challenges facing Africa and other regions in achieving SDG2 by 2030. The study will delve into the SDG2 plans and programmes of African nations and other countries, examining their implementation of the five key SDG 2 targets (5). The following section introduces the articles included in this Research Topic. The paper concludes with a summary of the key findings and overall insights.

## 2 Articles in the Research Topic

This Research Topic (RT) presents eight articles (six original research articles, one study protocol, and one review article) that have successfully undergone rigorous peer review, meeting the high standards of Frontiers in Public Health. While these articles explore diverse topics, theoretical perspectives, and methodologies, they are all connected to the overarching themes of this RT.

Despite numerous interventions aimed at promoting healthy diets and reducing food insecurity, limited research exists on the consumption patterns that contribute to achieving healthy diets and zero hunger within households, particularly the budget allocation toward specific food items. Ogunleke et al. investigated these patterns among 600 rice-consuming households in South-West Nigeria (Lagos, Osun, and Ogun states) using primary data. Their study revealed that rice, particularly local (Ofada) rice, is the most consumed food item in the region, accounting for a significant 19.5% of the monthly household budget. Employing a linear approximated QUAIDS model, the study analyzed household demand for local rice in the South-West region. Findings indicate that local rice consumption is substantial in this region and that local rice is not considered a luxury good based on the calculated expenditure elasticities, all of which are expenditure inelastic.

Adeyemo and Adeagbo's Nigerian study examined the impact of land reforms on smallholder farmers. The research utilized data from the 2018/2019 Living Standard Survey Integrated Survey on Agriculture (LSMS/ISA) involving 4,032 respondents. The findings showed that despite low land titling rates (around 12%), land titles had a significant positive impact on agricultural production. Households with land titles also had significantly higher food expenditures (N9,868) compared to those without titles (N6,172). The study concluded that implementing and expanding formal land registration and titling could be a critical step toward achieving food security goals in Nigeria.

Despite ongoing efforts, the global hunger crisis continues to worsen. Adding to this challenge is the widespread issue of food waste, which disrupts food supply chains and negatively impacts the environment due to inefficient waste management. Zaini et al.'s review article identifies key waste streams generated during the production of mushroom, peanut, and soybean (MPS). Mushroom, with its functional and nutraceutical properties, is increasingly recognized as a future food source. Peanuts, known for their rich nutritional value, are consumed globally and are a staple ingredient in many cuisines. Soybean, a significant plant-based protein source, is widely cultivated and consumed worldwide, particularly in Asia (8). The article highlights the critical potential of MPS as future foods in the fight against hunger, while also acknowledging the challenges that need to be addressed. Sawadogo et al. examined the factors contributing to the decline in chronic malnutrition in Burkina Faso during the 2000s, a period marked by increased access to healthcare services. Utilizing data from Demographic and Health Surveys (DHS) conducted in 2003 and 2010, the study analyzed the temporal variations in chronic malnutrition using the Oaxaca-Blinder multivariate decomposition method. Their findings revealed that improved access to healthcare services significantly contributed to the reduction of chronic malnutrition between 2003 and 2010. The study emphasizes the critical importance of ensuring access to healthcare services for children as a cornerstone of any programmes aimed at tackling child malnutrition.

Child and maternal malnutrition (CMM) remains a major global health challenge, contributing significantly to disability-adjusted life years (DALYs) and deaths. Liu et al., in a comprehensive analysis using data from the Global Burden of Disease study 2019 (GBD 2019), examined the global impact of CMM from 1990 to 2019. Their findings revealed a substantial burden, particularly affecting infants under 28 days old, with sub-Saharan Africa experiencing the most significant impact. Globally, low birth weight and short gestation were identified as primary risk factors for CMM. The study highlights the crucial influence of national economic levels, healthcare expenditures, and the allocation of medical resources on the disease burden associated with CMM.

Anemia remains a significant health concern among women of reproductive age in sub-Saharan Africa. Tirore et al. examined the prevalence and severity of anemia in 21 countries using recent Demographic and Health Survey (DHS) data from 2015 to 2022. Their analysis of 171,348 women revealed a concerning prevalence of anemia at 41.74%. The study identified several factors contributing to higher anemia risk, including distance from healthcare facilities, poverty, lack of improved sanitation, and the use of solid cooking fuel. The researchers emphasize the crucial need to integrate women's empowerment, through education and economic opportunities, into anemia prevention and control programmes across Africa.

Downs et al. have designed a study protocol to evaluate the efficacy of mHealth interventions in promoting recommended behavior change communication (BCC) strategies among caregivers of infants and young children in Senegal. The trial will take place in three regions (Thies, Fatick, Diourbel) and involve 488 mother-father-child triads, with children aged 6 to 23 months. The primary outcomes of interest are minimal acceptable diet (MAD) and anemia. The study will gather qualitative data through focus group discussions with mothers and fathers and semi-structured interviews with Badienou Gox and national partners and program implementers to assess the intervention's implementation process.

Undernutrition remains a major health concern in many developing countries, particularly affecting children in Africa, especially in Ethiopia. Yitayew et al. investigated the prevalence of acute malnutrition relapse among under-five children in Northeast Ethiopia. Their study, involving 318 children who had been enrolled and discharged from a community-based acute malnutrition management programme, revealed an alarming 35.2% relapse rate after discharge. The researchers emphasize the critical need for comprehensive health education and counseling services to improve maternal knowledge and practices, focusing on child immunization and hygiene maintenance to prevent diarrheal diseases, which can significantly contribute to reducing the risk of malnutrition relapse.

## 3 Conclusion

This Research Topic brought together eight articles that collectively address a critical gap in the literature by exploring global progress in combating hunger, with a particular focus on the African region. The articles analyse the interlinkages between SDG 2 (Zero Hunger) and other Sustainable Development Goals, while also examining the opportunities and challenges facing African nations and other regions in achieving Zero Hunger by 2030. The research delves into the implementation of relevant plans and programmes, offering valuable insights into the complex challenges and potential solutions in the pursuit of a hunger-free world.
